# Comparison of clinicoradiologic manifestation of nonspecific interstitial pneumonia and usual interstitial pneumonia/idiopathic pulmonary fibrosis: A report from NRITLD

**DOI:** 10.4103/1817-1737.43081

**Published:** 2008

**Authors:** Saeid Fallah Tafti, Bahareh Mokri, Foroozan Mohammadi, Mehrdad Bakhshayesh-Karam, Habib Emami, Mohammad Reza Masjedi

**Affiliations:** *Department of Internal Medicine, National Research Institute of Tuberculosis and Lung Disease (NRITLD), Shahid Beheshti University of Medical Sciences and Health Services MC, Shaheed Bahonar Ave., Darabad, Tehran 19569, P.O: 19575/154, Iran*; 1 *Department of Pathology, National Research Institute of Tuberculosis and Lung Disease (NRITLD), Shahid Beheshti University of Medical Sciences and Health Services MC, Shaheed Bahonar Ave., Darabad, Tehran 19569, P.O: 19575/154, Iran*; 2 *Department of Radiology, National Research Institute of Tuberculosis and Lung Disease (NRITLD), Shahid Beheshti University of Medical Sciences and Health Services MC, Shaheed Bahonar Ave., Darabad, Tehran 19569, P.O: 19575/154, Iran*

**Keywords:** Interstitial lung diseases, pneumonitis, tomography, X-ray, computed

## Abstract

**BACKGROUND:**

Ever since Katzenstein and Fiorelli introduced the term nonspecific interstitial pneumonia (NSIP) to denote those cases of interstitial pneumonia that cannot be categorized as any of the other types of idiopathic interstitial pneumonias (IIP), there has been continuing debate on whether it is a real clinical entity or not. The American Thoracic Society/European Respiratory Symposium task group tried to identify idiopathic NSIP as a separate disease and exclude it from the category of IIP. However, it appears that the clinical presentation of NSIP and usual interstitial pneumonia (UIP) are the same.

**OBJECTIVE:**

To show that the radiologic features of NSIP and UIP should be relied upon, instead of clinical presentation and pathologic findings, to differentiate between the two.

**MATERIALS AND METHODS:**

Consecutive patients who had received a diagnosis of either NSIP or UIP on the basis of open lung biopsy between January 2001 and December 2007 were identified for inclusion in this retrospective review. The study included 61 subjects: 32 men and 29 women with a mean age of 59.39 ± 14.5 years. Chest computed tomography images of all the cases were collected for a review. High resolution computed tomography (HRCT) and all pathologic specimens were also evaluated. A weighted kappa coefficient was used to evaluate whether radiology can be used instead of biopsy for the diagnosis of NSIP and UIP. Comparison of the mean ages and the time intervals (i.e., interval between symptom onset and the time of diagnosis) in the UIP and NSIP groups was done using the Mann-Whitney U test. Association between gender and biopsy result was evaluated by the Fisher exact test. Data were evaluated using SPSS, v.13.

**RESULTS:**

Sixty-one patients were included in this study, 32 were male and 29 were female. On the basis of biopsy findings, 50 (82%) patients had UIP and 11 (18%) had NSIP. Thirty (60%) of the 50 patients who had UIP were male and 20 (40%) were female; 2 (18.2%) of the 11 patients who suffered from NSIP were male and 9 (81.8%) were female. Based on HRCT findings, 36 (60%) patients were diagnosed to have UIP and 24 (40%) were diagnosed with NSIP. When diagnosis was based on biopsy findings, the time interval in the UIP group was 13.59 ± 8.29 months and in the NSIP group it was 7.90 ± 4.18 months. When diagnosed on the basis of HRCT findings, the time interval in the UIP group was 14.22 ± 8.94 months and in the NSIP group it was 10.54 ± 5.78 months. When diagnosis was on the basis of biopsy, the mean age in the UIP group was 61.30 ± 14.18 years and in the NSIP group it was 50.73 ± 13.14 years.

**CONCLUSION:**

HRCT can be used instead of invasive methods like lung biopsy to differentiate between UIP and NSIP.

Ever since Katzenstein and Fiorelli introduced the term nonspecific interstitial pneumonia (NSIP) for those cases of interstitial pneumonia that, because of the pathologic findings, cannot be categorized as one of the other types of idiopathic interstitial pneumonias (IIPs), there has been much debate on whether it is a real clinical entity or not. The American Thoracic Society/European Respiratory Symposium task group tried to classify idiopathic NSIP as separate disease and exclude it from the category of IIPs.

Several studies have shown that in addition to surgical lung biopsy, high resolution computed tomography (HRCT) has also assumed a great role in IIP diagnosis and management[1–3]; however, the ability of HRCT to distinguish between usual interstitial pneumonia (UIP) and NSIP is debated. Some recent reports suggest variable appearance of NSIP.[4,5]

Histologically NSIP with fibrosis is characterized by a temporal uniformity of the disease process with varying degrees of interstitial inflammation or fibrosis. However, the concept of a predominant inflammatory or cellular interstitial pneumonitis was proposed by Carrington *et al.* and, more recently, by Nagai and Kitaichi,[6–8] who termed the histopathologic pattern of unclassified interstitial pneumonia or cellular interstitial pneumonia as NSIP, so, NSIP is not a new pulmonary process.

NSIP may have various etiologies; for example, it may be idiopathic, a variant form of idiopathic pulmonary fibrosis (IPF), secondary to collagen vascular disease, drug induced, occupational, infectious, familial, due to chronic aspiration, or granulomatous. NSIP tends to be associated with collagen vascular disease.[9–11]

In general, patients with NSIP experience slowly progressive dyspnea. Other common symptoms include nonproductive cough, fatigue, malaise, anorexia, and weight loss; low grade fever has also been reported. Clubbing was reported in 10% of the patients with NSIP and in 65% of IPF cases, including UIP.[12,13] It appears that the clinical presentation of NSIP and UIP are similar.

Despite the definition of NSIP mentioned earlier, some similarity exists between NSIP, UIP, hypersensitivity pneumonia, and other IPF subtypes. UIP is characterized by patchy scarring of the lung parenchyma, with intervening normal or nearly normal alveoli. The most fibrotic zones show honeycombing, with complete destruction of the architecture and presence of inflammation in these regions. The subpleural and paraseptal distribution, the patchy character, and the temporal heterogeneity are the features that are most helpful in establishing the diagnosis of UIP.[14]

On the other hand, uniform involvement of lung parenchyma, marked chronic inflammation in the interstitium, and prominent pericentral scarring are all features that argue against the diagnosis of UIP, but are findings commonly observed in NSIP.[14]

Initially, NSIP was diagnosed when the histopathologic findings did not fit the classic patterns of other IIP subtypes.

Until now, surgical lung biopsy has been considered necessary for confirmation of diagnosis in all cases of IIP, including NSIP. In this study we would like to argue that the radiologic findings, as also the time interval and clinical presentation, can be used to differentiate between UIP and NSIP, and invasive methods such as biopsy can be avoided.

## Materials and Methods

Consecutive patients, who had received a diagnosis of rather NSIP or UIP on the basis of open lung biopsy between January 2001 and December 2007, were identified for inclusion in the study. The pathologists assigned a diagnosis of UIP, NSIP, or other to each case. The definition of NSIP was still evolving at the beginning of this study and therefore all specimens classified as NSIP were re-reviewed of the end of study period (December 2007) to confirm the accuracy of the initial diagnosis.

We identified 61 patients for inclusion in the study. There were 32 males and 29 females; the mean age was 59.39 ± 14.5 years. From this group of cases, all those who had had computed tomography (CT) of the chest done were included for review.

Fortunately, all 61 patients who participated in this study had chest x-ray and HRCT done. HRCT was done routinely before lung biopsy for all patients because biopsy sites were determined on the basis of HRCT findings.

CT was performed mainly with a high-speed advantage scanner. Thin-section CT scans with 1-mm collimation were obtained at 10-mm intervals with the patients in the supine position and at 20-mm intervals with the patients in the prone position. Images were reconstructed with high-special-frequency algorithm and graphies at window's setting were appropriated for viewing the lung parenchyma (window's center: 550HU, window's width 1,500HU). Two expert thoracic radiologists independently reviewed the HRCT scans. No clinical information was provided to them.

Surgical lung biopsies were performed by either open thoracotomy or video-assisted thoracoscopy. The surgeon was asked to obtain multiple biopsies from different sites and was able to achieve this in all the selected patients.

A pathologist, experienced in the field of lung diseases and blinded to the clinical and radiological features, reviewed the biopsy specimens. Each specimen was assigned a histological diagnosis of UIP or NSIP using the defined criteria.

Cases associated with connective tissue diseases, occupational exposure, exposure to drugs, infection, or those who had history of prior treatment with corticosteroids or immunosuppressants were excluded.

In accordance with the standard definitions, lung parenchymal abnormality was recorded as being predominantly ground-glass attenuation (group 1); predominantly reticular or mixed type (group II); or honeycombing, when there was a cluster of air spaces of more or less uniform diameter of 0.3–1.0 cm (group III).

A weighted kappa coefficient was used to evaluate whether radiology can be used instead of biopsy for diagnosis of NSIP and UIP. Comparison of ages and time intervals (i.e., the interval between symptom onset and the time of diagnosis) between the UIP and NSIP groups was evaluated by the Mann-Whitney U test. Association between gender and biopsy results were evaluated by the Fisher exact test. Data were analyzed using SPSS v.13.

## Results

Of the 61 patients included in the study, 32 were male and 29 were female. On the basis of biopsy findings, 50 (82%) patients suffered from UIP and 11 (18%) suffered from NSIP. Thirty (60%) of the 50 patients who had UIP, were male and 20 (40%) were female; 2 (18.2%) of the 11 patients who suffered from NSIP were male and 9 (81.8%) were female.

HRCT of 36 patients (60%) showed the honeycombing pattern and 24 patients (40%) had bilateral ground-glass and irregular reticular pattern.

In this study, 16 patients (26.2%) had been referred to our institute because of dyspnea, 4 (6.6%) because of cough, and 41 (67.2%) because of both dyspnea and cough. On examination, clubbing was seen in 39 (75%) patients, crackles were detected in 8 (15.4%), and both crackles and clubbing were present in 5 (9.6%) patients.

When diagnosis was by biopsy, the time interval between onset of symptoms and diagnosis of UIP was 13.59 ± 8.29 months, whereas the time interval was 7.90 ± 4.18 months in cases of NSIP; in comparison, when diagnosis was based on HRCT, the time interval in cases of UIP was 14.22 ± 8.94 months and in NSIP it was 10.54 ± 5.78 months. When diagnosis was made on the basis of biopsy findings, the mean age of patients with UIP was seen to be 61.30 ± 14.18 years, while it was 50.73 ± 13.14 years for patients with NSIP. The kappa coefficient value was 0.50, which is in the moderate range [Table 1].

**Table 1:** Demographic characteristics of 61 patients with usual interstitial pneumonia and nonspecific interstitial pneumonia

	Usual interstitial pneumonia	Nonspecific interstitial pneumonia
Number	50 (82)	11 (18)
Sex		
Male	30 (60)	2 (18.2)
Female	20 (40)	9 (81.8)
Age (mean ± SD)	61.30 ± 14.18	50.73 ± 13.14
Symptom		
Dyspnea	9 (18)	5 (45.5)
Cough	9 (18)	2 (18.2)
Both	33 (66)	4 (36.4)
Sign		
Clubbing	42 (84)	3 (27)
Crackle	47 (94)	10 (91)
HRCT		
Bilateral ground-glass	35 (70)	10 (90.9)
Irregular reticular pattern	9 (18)	4 (36.3)
Honeycombing in subpleural and lower lung zone	41 (82)	1 (9)
Time interval (months)		
In biopsy	13.59 ± 8.29	7.90 ± 4.18
In HRCT	14.22 ± 8.94	10.54 ± 5.78

Figures in parentheses are in percentages.

## Discussion

After several meetings, a multidisciplinary panel has concluded, and several studies have also shown, that the accuracy of diagnosis of IPF is high when the clinical and radiologic features are entirely consistent with ‘no unusual features’ seen. Also, the diagnosis of NSIP without biopsy is highly inaccurate, with roughly 50% of cases being missed. HRCT has an important diagnostic role in IIP.[15] We hypothesized that the HRCT appearance would have an impact on differentiation between NSIP and UIP when the degree of confidence in the diagnosis is high.[16,17] We found that although the honeycombing pattern is more common in CTs of UIP patients, some of the NSIP patients may also have a honeycombing pattern in their CTs. This finding is supported by a few articles that have indicated that UIP can be diagnosed on the basis of honeycombing, as this finding correlates strongly with pathological fibrosis and impaired survival.[18–20] The absence of a honeycombing pattern, the presence of ground-glass opacity, and an apical or non-subpleural distribution confirms the diagnosis of NSIP.[21]

In our study, we found that the predominant histological features of NSIP are interstitial inflammation and fibrosis. An interesting point noticed when the two groups are compared is that the mean age of NSIP patients was significantly lower than that of UIP patients.

With respect to age and sex differences between UIP and NSIP, other studies have shown that men are more likely to suffer from UIP and women from NSIP. In addition, the mean age of NSIP patients has been reported to be lower than that of UIP patient. These studies have reported that the signs and symptoms in NSIP and UIP are almost the same but mode of onset of NSIP is lower than UIP.[22,23] These findings are similar to ours: we too found that the time between symptom onset and diagnosis (which we termed the time interval) is less in the NSIP group than in the UIP group. Also, our study confirmed that UIP was more often seen in men, whereas NSIP was more common in women; and the signs and symptoms are approximately the same in both the sexes.

**Figure 1:**
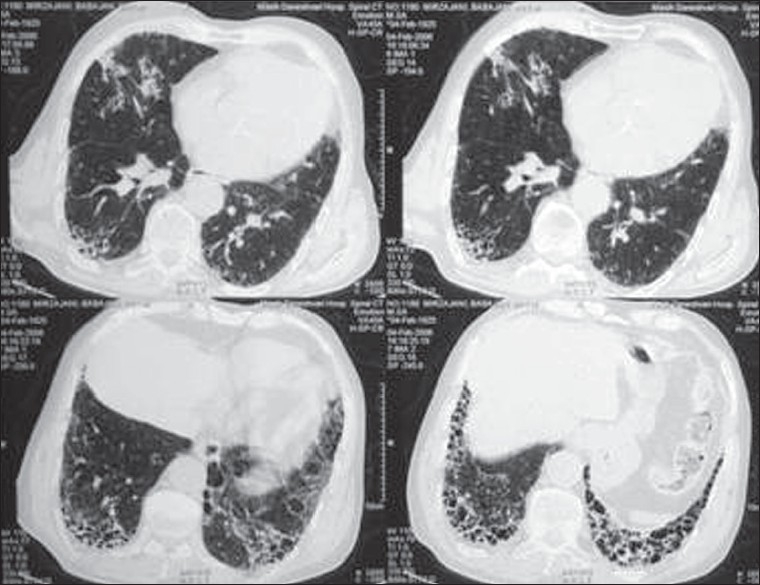
CT scans in usual interstitial pneumonia

**Figure 2:**
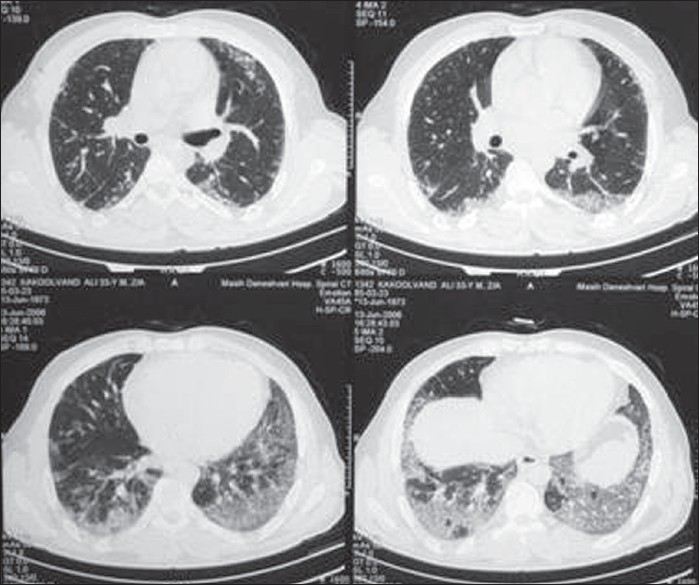
CT scans in nonspecific interstitial pneumonia

In this study, although the mode of onset of UIP was seen to be different from that of NSIP, the differences between the symptoms or signs were not readily apparent. The most significant finding in HRCT was the presence of bilateral patchy areas of ground-glass opacity, frequently accompanied by areas of consolidation and irregular linear opacities in the lower zone distribution. NSIP could be differentiated from UIP by the lack of subpleural honeycombing in the former, the most characteristic difference between the two diseases [Figures 1and 2].

Some studies have reported that ground-glass attenuation is the salient feature of NSIP, being found in 76–100% of cases, which is consistent with our findings. The ground-glass attenuation is frequently associated with traction bronchiectasis, suggesting the occurrence of lung fibrosis. Associated reticular abnormalities are found in 46–93% of NSIP cases; the prevalence of honeycombing ranges from 0–30%[23,24] and the prevalence of consolidation ranges from 16–80% in NSIP cases.[25]

Although some previous studies had suggested specific HRCT findings in patients with NSIP, more recent data has highlighted the limitations of these radiographic findings in diagnosis.[26] The presence of a predominant ground-glass attenuation pattern in CT was probably due to the presence of histologic NSIP and, therefore, was associated with a greater likelihood of response to steroid treatment.

In inspections, several studies have confirmed that this entity, now called NSIP, is associated with a substantially better prognosis than UIP.[27–29] NSIP is recognized as one of the most common histological findings in patients with IIPs—less common than UIP but much more common than desquamative interstitial pneumonia (DIP). NSIP is subclassified into cellular and fibrotic types.[29]

Cellular NSIP is less common than fibrotic NSIP and it carries a substantially better prognosis, with excellent survival at 5 and 10 years.[29] Cellular NSIP was found to be associated with a finer pattern of fibrosis and a lower likelihood of subpleural distribution as compared with fibrotic NSIP.

After treatment with corticosteroids, the abnormalities of NSIP seen on CT completely or partially resolve. In patients with UIP, the concept that the ground-glass attenuations represent potentially reversible alveolitis is no longer valid, because alveolar inflammation is not recognized as a significant part of the histology of UIP.[30]

Indeed, after excluding cases of NSIP and other more benign entities, it is apparent that UIP has a worse prognosis than many types of cancers, with a low likelihood of response to steroids and a median survival of 2.2-8 years after diagnosis.[30]

The presence of a predominant ground-glass pattern in CT scans is probably due to the presence of histologic NSIP, and the accompanying areas of consolidation that are frequently seen reflect the underlying interstitial inflammation and, therefore, may be associated with a greater likelihood of response to treatment. Active inflammation of the lung parenchyma induces clinical symptoms in a subacute fashion, whereas in UIP the converse is true and the natural history is chronic and progressive and is associated with greater than 60% mortality.[31,32]

Furthermore, on the basis of overlap of CT appearances in IPF and NSIP, radiologists must adhere to strict CT criteria for establishing this dismal diagnosis.[33]

Up to now, an invasive method such as lung biopsy was more used than other methods such as HRCT for the diagnosis of NSIP and UIP. The present study has revealed that differentiation of NSIP from UIP is feasible by HRCT, and invasive methods are not needed.

On the basis of our findings and that of studies, we also advice that to differentiate between NSIP and UIP or other types of IIP the focus should be on age, time interval between symptom onset and diagnosis, and HRCT findings instead of symptoms, signs, and pathologic features. This strategy, in patients with respiratory symptoms that are suspected to be due to NSIP or UIP, can help us to avoid an invasive procedure like open lung biopsy.

Finally, according to Riha and Nagai *et al.*[11,22] the honeycombing pattern was found in HRCTs of NSIP patients. We suggest that this may have been because UIP often coexists with NSIP and, hence, a small proportion of HRCTs in NSIP patients shows the honeycombing pattern. Therefore, NSIP cannot be considered an independent disease as yet.

Coexistence of histologic patterns is suggestive of NSIP and UIP in the same patient. Histologic heterogeneity has received little attention in the literature because more biopsy from multiple lobes is needed to establish this.[34–36]

Our study is limited by the small sample size and by the fact that it is a retrospective review. In studies of this type there can be considerable selection bias and there may have been inconsistencies in the interpretation of the histologic findings. Although, we tried to include only patients with newly diagnosed disease, it is possible that at the time that imaging was done our population included both treated and untreated patients.

More studies with larger samples are needed to confirm if biopsy can be replaced completely by HRCT or not. Biopsy sometimes fails to differentiate between NSIP and UIP and diagnosis may be more accurate by HRCT.

In summary, it is possible to use HRCT instead of lung biopsy to diagnose NSIP and UIP. Also of help in arriving at a correct diagnosis are factors such as the differences in the time interval between symptom onset and diagnosis, mean age, and sex.
